# Simultaneously Enhancing the Flame Retardancy, Water Resistance, and Mechanical Properties of Flame-Retardant Polypropylene via a Linear Vinyl Polysiloxane-Coated Ammonium Polyphosphate

**DOI:** 10.3390/polym15092074

**Published:** 2023-04-27

**Authors:** Qining Ke, Junchen Bai, Ge Zhang, Jiacheng Zhang, Mingshu Yang

**Affiliations:** 1 Beijing National Laboratory for Molecular Sciences, Key Laboratory of Engineering Plastic, Institute of Chemistry, Chinese Academy of Sciences, Beijing 100190, China; keqining@iccas.ac.cn (Q.K.); junchenbai@iccas.ac.cn (J.B.); zhangge20@iccas.ac.cn (G.Z.); zhangjc717@iccas.ac.cn (J.Z.); 2 University of Chinese Academy of Sciences, Beijing 100049, China

**Keywords:** polypropylene, ammonium polyphosphate, vinyl polysiloxane, flame retardancy, water resistance

## Abstract

It is challenging to improve the water resistance, flame retardancy, mechanical performance, and balance of halogen-free flame-retardant polypropylene (PP) composites. For this purpose, a linear vinyl polysiloxane (PD) was synthesized and then self-crosslinked under benzoyl peroxide to prepare surface-coated ammonium polyphosphate (APP@PD). Apparently, this linear vinyl polysiloxane self-crosslinking coating strategy was completely different from the commonly used sol-gel-coated APP with silane monomers. After coating, the water contact angles (WCA) of APP and APP@PD were 26.8° and 111.7°, respectively, showing high hydrophobicity. More importantly, PP/APP@PD/dipentaerythritol (DPER) showed a higher limiting oxygen index (LOI) and better UL-94 V-0 rate in comparison with PP/APP/DPER composites. After water immersion at 70 °C for 168 h, only PP/APP@PD/DPER kept the UL-94 V-0 rate and lowered the deterioration of the LOI, reflecting the better water-resistance property of APP@PD. Consistently, the cone calorimeter test results displayed a 26.2% and 16.7% reduction in peak heat release rate (PHRR) and total smoke production (TSP), respectively. Meanwhile, the time to peak smoke production rate (T_PSPR_) increased by 90.2%. The interfacial free energy (IFE) between APP@PD and PP was calculated to evaluate the interfacial interaction between PP and APP@PD. A reduction of 84.2% in the IFE between APP@PD and PP is responsible for the improvement in compatibility and the increase in flame retardancy, water resistance, and mechanical properties of the composites.

## 1. Introduction

Owing to the extensive applications of polypropylene (PP), significant efforts have been made during the past decades to improve its flame retardancy [1,2]. Halogen flame retardants exhibit promising flame retardancy, but the high production of toxic smoke during combustion leads to damage to the human body and environment. Hence, with the increasingly stringent use conditions of polymeric materials, environmentally friendly, cost-effective, and highly efficient flame retardants have necessarily been demanded. Intumescent flame retardants (IFRs) have been suggested as promising substitutes for the widely used halogen-containing flame retardants [3,4,5]. IFRs, mostly including ammonium polyphosphate (APP), show low toxicities, acceptable costs, and outstanding smoke suppression. Unfortunately, the intrinsic polarity of APP causes poor compatibility with PP, finally leading to the deterioration of the properties of the composites. Apart from that, its high moisture-sensitivity characteristics and poor compatibility with the polymer matrix can make it migrate out of the composites when they are at high temperatures or immersed in water, resulting in dramatically reduced flame retardancy and deterioration of the surface appearance of the materials. Therefore, the modification of APP may be one of the key means of enhancing the performance of IFRs.

Generally, microencapsulation or surface coating seems to be one of the most effective modification methods for APP in the use of flame-retardant polymer materials. The wettability, polarity, thermostability, flame retardancy, etc. of APP could be adjusted via the coating process. Melamine-formaldehyde resin [6,7,8,9,10,11], epoxy resin [12], phenolic resin [13,14], and polyurethane resin [15,16,17,18] were the commonly used shell materials of APP in the past decades. For most of the above-mentioned coatings of APP, although the wettability, polarity, and compatibility are improved, it is still hard to reconcile different properties. For instance, high flame retardancy, mechanical performance, and other properties. Thus, finding new coating methods or materials for APP is still necessary.

Polysiloxane, which is an organic–inorganic polymeric compound, has recently been used as a shell material of APP. This is because silicon particles immigrate to the surface of the materials during the burning process and establish a stable protective barrier that enables better flame retardancy in materials [19]. Attempts to prepare crosslinked silane coating APP via a sol-gel method with Vinyltriethoxysilane [20], vinyltrimethoxysilane [21], tetraethoxysilane [22], aminopropyltriethoxysilane [23,24], etc. [25,26] have been undertaken. They proved that the crosslinked silane coating possessed good thermal stability, hydrophobicity, and chemical durability. However, the flame retardancy was still unsatisfactory. Recently, polysiloxane with mainly organic groups has been directly added into the polymer matrix as a synergist component to improve the flame retardancy [27,28,29,30,31]. As a result, the flame retardancy and toughness of the composites were improved. As opposed to the main inorganic structure of the crosslinked silane obtained by the sol-gel method, the polysiloxane with numerous hydrocarbon groups could be catalyzed by phosphoric acid to reflect a better synergistic effect in the condensed phase of flame-retardant polymers [27,32,33]. Meanwhile, the mechanical performance may also improve. Unfortunately, using polysiloxane directly as a raw coating material for APP has not been tried in the literature; thus, we believe it will be interesting to coat APP with polysiloxane.

In this work, a vinyl polysiloxane (PD) was first synthesized, then simultaneously crosslinked and coated on the surface of APP. The structure of the microencapsulation and its characteristics were determined by X-ray photoelectron spectroscopy (XPS), Fourier transform infrared (FTIR), scanning electron microscopy (SEM), water contact angle (WCA), and thermogravimetric analysis (TGA) tests. The flame retardancy, water resistance, combustion behavior, thermal stability, and mechanical performance of the PP composites were investigated. The flame-retardant mechanism of the PP composites and the interfacial interaction between APP and PP before and after the coating were further investigated.

## 2. Materials and Methods

### 2.1. Materials

PP (pellets), T30S, was purchased from China Hohhot Petrochemical Co., Ltd., with a melt flow index of 3.0 g 10 min^−1^ (Hohhot, Inner Mongolia, China). APP (polymerization degree: *n* > 1000) and benzoyl peroxide (BPO) were bought from Shanghai Aladdin Biochemical Technology Co., Ltd. (Shanghai, China). Tetramethyltetravinylcyclotetrasiloxane (D4-VI), tetramethylammonium hydroxide pentahydrate (Me4NOH), 1,3-divinyltetramethyldisiloxane, and dipentaerythritol (DPER) were obtained from Beijing InnoChem Science & Technology Co., Ltd. (Beijing, China). Anhydrous ethyl alcohol and dimethyl sulfoxide (DMSO) were obtained from Beijing Chemical Reagent Company (Beijing, China). The antioxidant B215 was acquired from Ciba Specialty Chemicals Co., Ltd. (Shanghai, China Division). The deionized water was provided by the Institute of Chemistry Chinese Academy of Sciences (Beijing, China). All reagents were used without further treatment.

### 2.2. Synthesis of PD

**Scheme 1:**
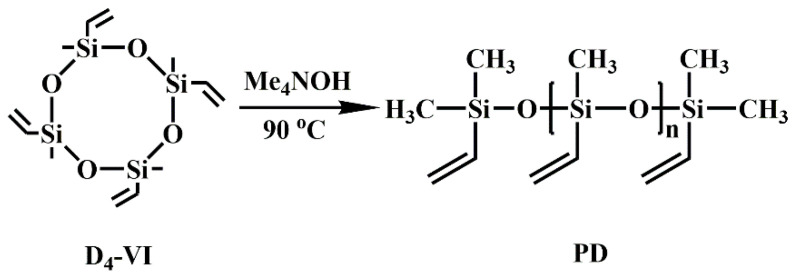
Synthesis process of PD.

Polymeric D4-VI (PD) was synthesized via the ring-opening homo-polymerization of D4-VI. Firstly, D4-VI (0.1 mol, 34.47 g), 1,3-divinyltetramethyldisiloxane (0.01 mol, 1.86 g), and DMSO (16.4 µL, 0.05 wt.%, as a catalyst solubilizer) were sequentially added to a three-necked flask under vigorous stirring and at room temperature. Then, the atmosphere in the flask was replaced with nitrogen after 15 min, and a steady nitrogen flow was provided to protect the reaction from the air. Afterwards, tetramethylammonium hydroxide pentahydrate (Me4NOH, 0.03 wt.%) was added rapidly as a catalyst, and the reaction mixture was quickly heated to 90 °C and maintained for 8 h. After holding continuous stable stirring for 8 h at 90 °C, the raw product was obtained, and it was purified by evaporation (−0.1 MPa) for another 2 h to remove the unreacted monomers, oligomers, and catalyst at 135 °C (which is a little higher than the degradation temperature of the catalyst). The schematic representation of the synthesis process of PD is shown in Scheme 1.

### 2.3. Preparation of APP@PD

**Scheme 2:**
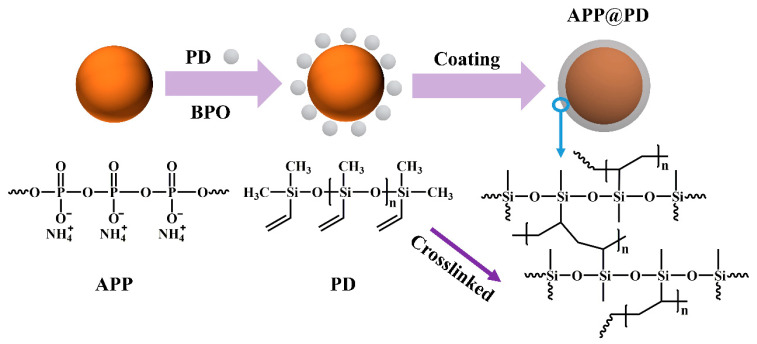
Synthesis process of APP@PD.

The modified APP (APP@PD) was synthesized via a crosslinking process of PD on the surface of the APP. Firstly, 32.0 g APP powder was evenly dispersed in 100 mL ethyl acetate in a three-necked flask under vigorous stirring at room temperature. Then, 8.0 g PD was added dropwise to the flask containing the reaction mixture. Afterwards, 0.24 g BPO was used as a catalyst, and the system was quickly heated to 90 °C and refluxed for 6 h. The vinyl and methyl groups in PD then crosslinked each other on the surface of the APP. Afterwards, the ethyl acetate was evaporated completely from the mixture after 2 h at 100 °C. Finally, the acquired solid product was washed with deionized water three times to eliminate the unmodified APP. The schematic representation of the synthesis process of APP@PD is shown in Scheme 2.

### 2.4. Preparation of the Flame-Retardant PP Composites

The PP composites were prepared via melt blending using a Haake Polylab OS RheoDrive 7 internal mixer (Thermo Fisher Scientific, Bremen, Germany) at 190 °C with a rotor speed of 10 r/min for the first 1 min and another 7 min at 50 r/min. The formulas of all the composites are displayed in Table S1. The composites were marked as PP/X_1_ (A/B), where X_1_ represents the contents (wt.%) of all the IFRs, and A and B refer to the different components of the IFRs. After melt blending, the UL-94 and LOI test specimens were obtained by injection moulding at 210 °C and under a pressure of 800 bar on a Haake minjet device (Thermo Fisher Scientific, Germany). The cone calorimeter test samples were acquired after a hot-pressing process under 5 MPa for 6 min at 200 °C.

### 2.5. Characterization

Fourier transform infrared (FTIR) spectra were acquired from a Nicolet 6700 spectrophotometer (Nicolet Instrument Company, Madison, WI, USA). The samples were well ground with KBr and the testing wavenumber range was from 4000 to 650 cm^−1^.

The molecular weight of PD, as well as its distribution, were performed via a high-performance liquid chromatography equipment utilizing tetrahydrofuran as the dissolving (3 μL/mL) and testing solvent.

The morphologies of the sample were observed by a scanning electron microscope (SEM, SU8020, Hitachi, Tokyo, Japan) under an accelerating voltage of 5 kV and a current of 10 μA.

X-ray photoelectron spectroscopy (XPS) spectra were analyzed by ESCALab220i-XL (VG Scientific, West Sussex, UK) with Al Kα rays. The thickness of testing sheets was less than 0.5 mm, and their width and length were about 3.0 mm.

Thermogravimetric analysis (TGA) was recoded using a Pyris 1 (Perkin-Elmer, Waltham, MA, USA) thermogravimetric instrument via a programmed heating process from 50 °C to 800 °C under a nitrogen flow of 20 mL·min^−1^ and a constant heating rate of 20 °C·min^−1^. The sample weight was 2–5 mg. Of these, PD was liquid, APP and APP@PD were fine powders, and the composites were pellets.

Water contact angle (WCA) measurement was operated on an OCA 15EC (DataPhysics, Filderstadt, Germany) instrument. Furthermore, in addition to the high cost of commercial contact angle goniometers, the contact angle goniometers that perfectly work with either USB microscopy cameras or other suitable equipment can also accurately characterize the surface wettability of solids [34,35]. Each sample was tested for at least 3 reliable points to calculate the final results.

The solubility of samples in water was tested as follows: 10 g power was vigorously stirred in 100 mL denoised water for 2 h at 25 °C, 50 °C, and 75 °C, respectively. The suspension was filtered and then centrifuged at a speed of 10,000 rpm for 20 min. Finally, 50 mL supernatant was dried to a constant weight at 80 °C, which allowed for the solubility of the samples to be obtained.

The UL-94 vertical burning test was conducted on a CZF-5 instrument (Jiangning Analysis Instrument Company, Jiangning, China) according to ASTM D 3801. The dimensions of the specimen were 80.0 × 13.0 × 3.2 mm^3^.

The limiting oxygen index (LOI) test was performed according to ASTM D 2863 on a Fire Testing Technology instrument (FTT, West Sussex, UK). The sample size was 80.0 × 10.0 × 4.0 mm^3^.

The water resistance test of specimens was executed according to UL 746C. The samples were immersed in water at 70 °C for 168 h. After the boiling, the samples were dried at 80 °C to a constant weight.

Cone calorimeter test (CCT) was measured on a cone calorimeter equipment (FTT, UK), which applied ISO 5660-1: 2015. All samples (100 × 100 × 3 mm^3^) were burned at a heating power of 50 kW·m^−2^.

The notched Izod impact test was performed at room temperature using an XJC-25D impact tester (Chengde Precision Testing Machine Co., Ltd, Chengde, China) according to GB/T 1843-2008.

The tensile performance was tested according to ASTM D412 on a mounted materials testing system (Instron 3365, Buckinghamshire, UK). The crosshead speed was 50 mm/min.

## 3. Results and Discussion

### 3.1. Characterization of PD and APP@PD

**Figure 1:**
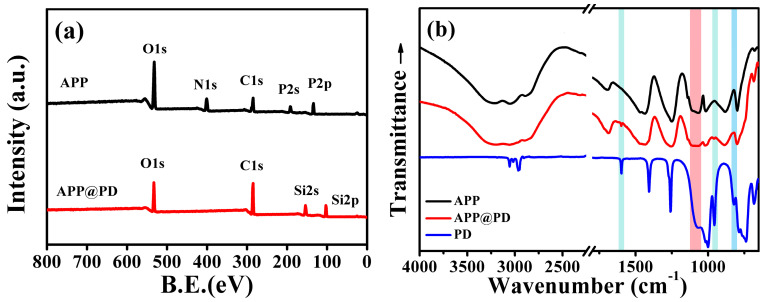
XPS (**a**) and FTIR (**b**) spectra of APP and APP@PD.

Generally, the FTIR spectra and TGA curves of PD are displayed in Figure S1. Compared with the spectra of D4-VI, the peaks at 1000–1100 cm^−1^ attributed to the Si–O−Si group of PD became split and smooth, proving its polymeric characteristics. Furthermore, PD exhibited good thermal stability with a residue of 68.9% at 700 °C, which is represented in Figure S1b. However, the boiling point of D4-VI was around 110 °C, as the material safety data sheet suggested. Furthermore, the Mn and Mw of PD were 6152 g/mol and 8144 g/mol, respectively, and it had a narrow distribution with a PDI of 1.33. To ensure the successful modification of APP, the XPS and FTIR spectra of APP and APP@PD were obtained. From what has been shown in Figure 1a and Table S2, after the coating, the new Si_2p_ and Si_2s_ peaks appeared while the N and P peaks disappeared simultaneously, proving that PD was successfully coated on the surface of APP. Similarly, according to the FTIR spectra of APP@PD in Figure 1b, the peaks around 1598 cm^−1^ and 961 cm^−1^ were ascribed to the stretching and bending vibrations of the remaining –CH = CH_2_ group. The peak at 1063–1116 cm^−1^ became smoother and wider, which could be attributed to the overlap between the Si–O and P–O groups. In addition, the peak at 825 cm^−1^ in the spectra of PD was ascribed to the bending vibration of Si–CH_3_, and the disappearance of that peak in the spectra of APP@PD suggested its crosslinking shell structure.

**Figure 2:**
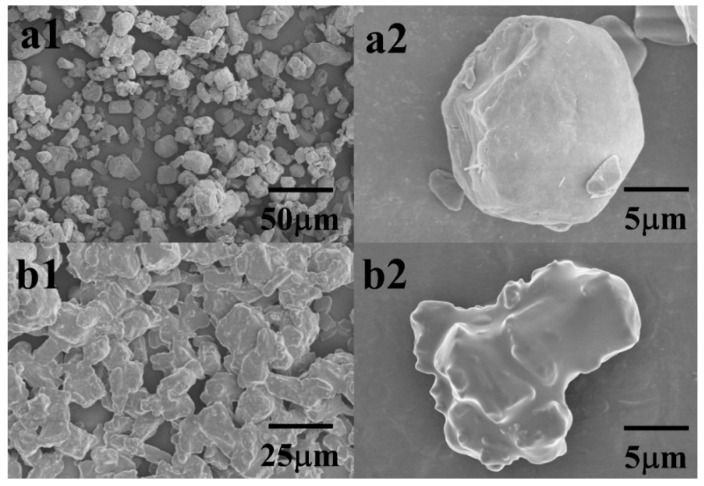
SEM micrographs of APP (**a**) and APP@PD (**b**).

The SEM micrographs of APP and APP@PD are presented in Figure 2. Evidently, edges and corners were easily observed from the surface of the APP. After the modification, a coating was obviously encapsulated on the surface of the APP, which made the surface of the particles smoother and allowed the crosslinking structures to be detected among some particles.

**Figure 3:**
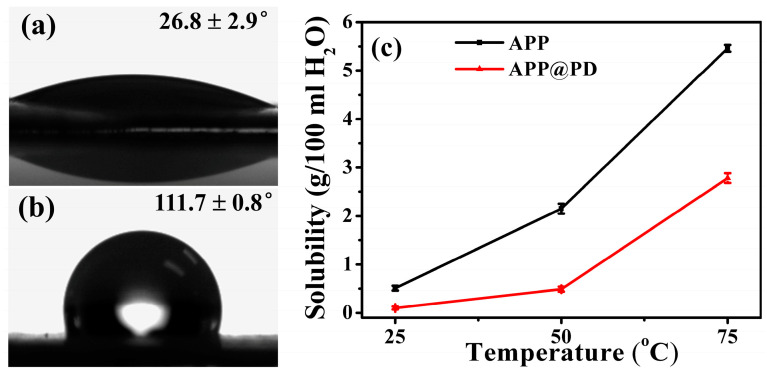
Water contact angle of APP (**a**) and APP@PD (**b**) and their water solubility at different temperature (**c**).

The water contact angle (WCA) and solubility of APP and APP@PD are shown in Figure 3. From the results shown in Figure 3a,b, the WCA of APP was 26.8°, indicating a high hydrophilicity. After the surface modification, APP@PD had a much bigger WCA of 111.7°. Furthermore, Figure 3c represents the changes in the water solubility of APP before and after the coating. The water solubility of APP at 25 °C, 50 °C, and 75 °C was 0.51, 2.15, and 5.46 g/100 mL, while that of APP@PD decreased to 0.10, 0.49, and 2.78 g/100 mL, respectively. Furthermore, the dispersion of APP and APP@PD in water was reflected in Figure S2. After stirring vigorously for 1 h, the APP@PD could easily sink after resting for 1 h, while some APP was still well dispersed in the water.

**Figure 4:**
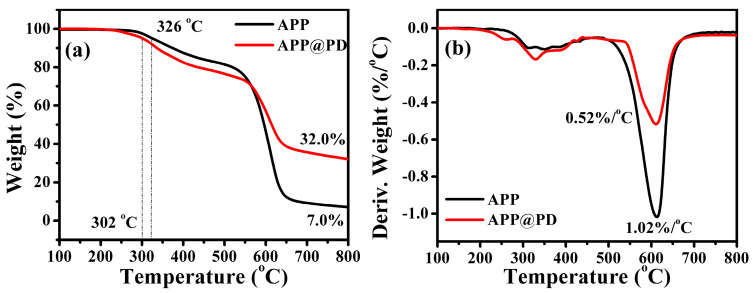
TGA (**a**) and DTG (**b**) curves of APP and APP@PD under nitrogen atmosphere.

Figure 4 displays the TGA (a) and DTG (b) thermograms of APP and APP@PD under a nitrogen atmosphere, from which it is seen that APP@PD, as distinct from APP, performed well in terms of enhanced thermal stability. Specifically, the residue (at 800 °C) was increased from 7.0% to 32.0%, accompanied by a dramatic decrease in the maximum weight loss rate from 1.02 to 0.52 %/°C. The initial decomposition temperature (at 5 wt.% weight loss) moved from 326 °C towards 302 °C, which might be attributed to the degradation of the PD chain (cf. Figure S1) and the crosslinking behavior between phosphorus and silicon in the condensed phase [19].

### 3.2. Flame Retardancy of the PP Composites

The LOI and vertical burning test results of the PP composites are listed in Table 1. Despite all the PP composites achieving the UL-94 V-0 rate under 25 wt.% IFR loading, only the PP/APP@PD/DPER composites attained the UL-94 V-0 rate when the loading decreased to 19 wt.%, as well as a 2.0% increase in LOI. While simply incorporating APP@PD into PP, a 5.1% increase in LOI was found. Apparently, outstanding synergistic behavior definitely existed among the crosslinked PD shell and APP.

The flame retardancy after the water immersion test of the sample is also shown in Table 1. After the water treatment, the LOI and UL-94 rating of PP/APP/DPER deteriorated dramatically. Nevertheless, the PP/APP@PD/DPER composites maintained the V0 rating (25 wt.% additive loading), and a lower ΔLOI was also detected, proving much better flame retardancy and water resistance.

**Figure 5:**
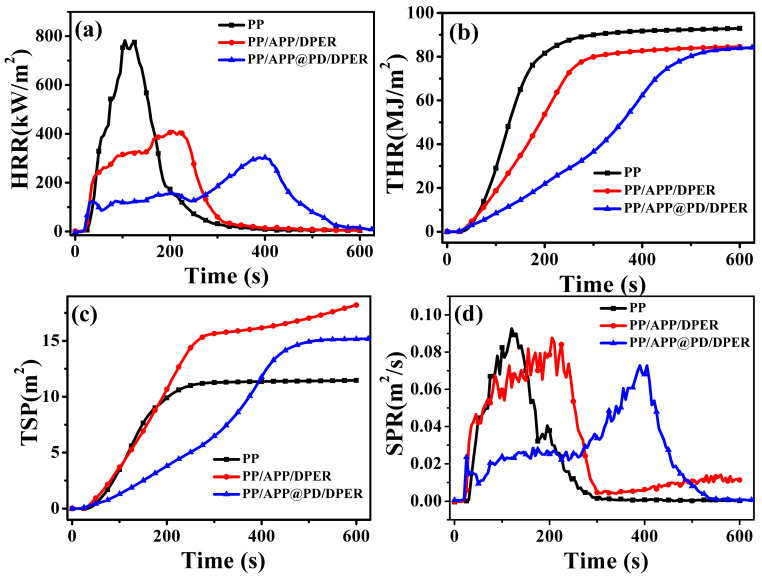
HRR (**a**), THR (**b**), TSP (**c**), and SPR (**d**) curves of PP and its composites.

CCT was applied to further evaluate the combustion behaviors of polymers. Figure 5 shows the heat release rate (HRR), total heat release (THR), total smoke production (TSP), and smoke production rate (SPR) curves of neat PP and PP composites as a function of combustion time. Obviously, the PP/APP@PD/DPER composite has lower HRR, peak HRR (PHRR, decreased by 26.2%), TSP (decreased by 16.7%), SPR, and peak SPR (PSPR) than those of the PP/APP/DPER composite. Furthermore, as the data given in Table S3 shows, after the coating of APP, the time to peak of SPR (T_PSPR_) was dramatically put off from 205 to 390 s (+90.2%), representing enhanced heat and smoke release suppression performance.

Distinctly, there was no difference in the THR and time to ignition (TTI) of the PP flame retardant composites. This could be attributed to the abundant flammable hydrocarbon groups in PD [29,36], which may contribute to the further burning of the material, and the similar av-EHC (Table S3) proved this as well. It can be seen that the TSP of pure PP was the lowest, which was related to incomplete combustion of the flame-retardant composites. In any case, the deteriorating effects outperformed the benefit from the enhancing char [37]. Furthermore, it is worth noticing that further smoke release was found in the PP/APP/DPER composites (>400 s). Indeed, this is a normal phenomenon in IFR systems [16,38,39]. The decomposition of the char on the surface of the materials [16] or the crack of the formed char layer [38] caused the further smoke release, showing an unstable char. In contrast, no further smoke release was found in the PP/APP@PD/DPER composite, suggesting a much more stable char. The digital photos displayed in Figure S3 after the CCT confirmed the enhancing charring ability.

### 3.3. Thermal Stability of the PP Composites and Analysis of Their Char Residues

**Figure 6:**
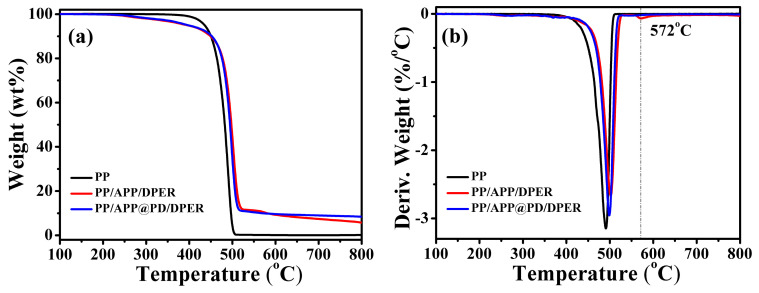
TGA (**a**) and DTG (**b**) curves of the PP, PP/APP/DPER, and PP/APP@PD/DPER composites under nitrogen atmosphere.

TGA was used to confirm the thermal stability of the PP composites. Figure 6 and Table 2 display the TGA and DTG results of PP and its composites under a nitrogen atmosphere. There is no distinct variation in the initial and maximum decomposition temperatures of the composites before and after the APP coating. However, it can be seen that the residues of the PP/APP/DPER and PP/APP@PD/DPER composites at 600 °C were almost the same. Nevertheless, a decomposition peak of the PP/APP/DPER composite appeared around 572 °C, as suggested by the DTG curves, and the residue showed a decrease to 5.7% when the temperature was elevated to 800 °C, representing an unstable residue formation. In comparison, the PP/APP@PD/DPER composite showed no degradation peak in this temperature range, indicating improved thermal stability of the residue.

**Figure 7:**
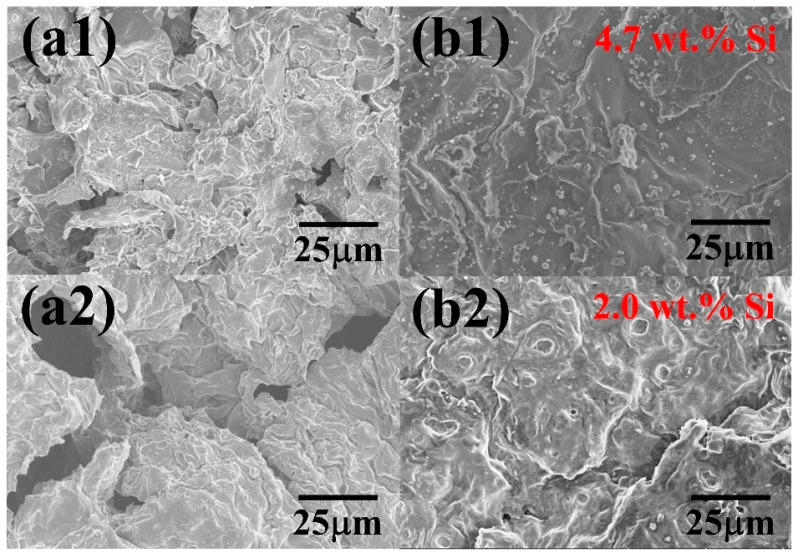
SEM micrographs of the char of the PP composites collected from the CCTs, (**a**): PP/APP/DPER, (**b**): PP/APP@PD/DPER. (“**1**”: outer surface of the char, “**2**”: inner surface of the char).

The SEM micrographs of the char from CCTs were carried out further to acquire more details about their stability. As is presented in Figure 7, the PP/APP@PD/DPER composite showed compact and continuous char, whereas the char of the PP/APP/DPER revealed a porous structure. Consequently, a stable char was obtained after the coating of APP. Moreover, the Si content of the char was higher on the outer surface than on the inner surface, revealing the migration of the Si element during the combustion process. The abundant silicon-containing compounds on the surface then prevented the formation of cracks in the char.

**Scheme 3:**
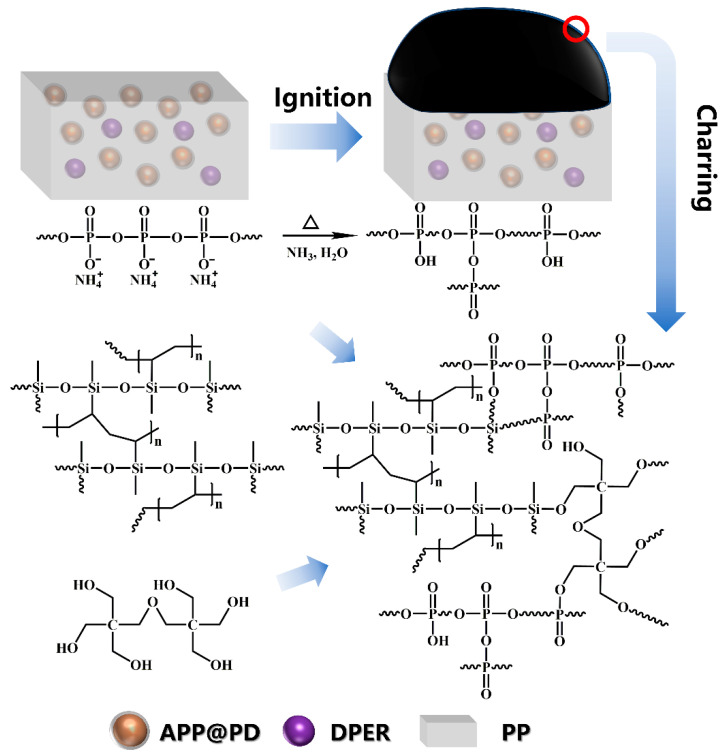
Possible flame-retardant mechanism of the PP/APP@PD/DPER composites.

Moreover, the FTIR spectra of the char are investigated. As shown in Figure S4, for PP/APP/DPER, the peaks located at 1163 cm^−1^ and 993 cm^−1^ indicated the stretching vibrations of C–O–C and C–O–P, respectively. For PP/APP@PD/DPER, the new overlapping peaks emerged at 1110–1180 cm^−1^ (three peaks) and 993–1037 cm^−1^ (two peaks) corresponded to the stretching vibrations of Si–O–P, Si–O–C, C–O–C, and Si–O–Si, C–O–P, respectively. According to the aforementioned results, a possible flame-retardant mechanism was proposed (Scheme 3). Since the flame prohibition effect of PP/IFR composites is well understood in the condensed phase, the enhanced flame-retardant mechanism of the PP/APP@PD/DPER composite is proposed as the following: The polysiloxane chain was introduced to enhance char formation and stability during the burning process. Firstly, the PD coating was catalyzed by APP to form the Si–O–P bond, and the appearance of the C–O–Si bond (cf. Figure S4) validated the reaction between PD and DPER. Then, a silicon-containing crosslinking structure stabilized the char when the temperature was high enough (cf. Table 2). During this period, the silicon compounds will immigrate to the surface of the char to suppress the heat transfer (cf. Figure 7). Overall, the flame retardancy of the composites was greatly improved by a more stable condensed phase during the combustion process.

### 3.4. Immigration of Additives during the Water Immersion Test

**Figure 8:**
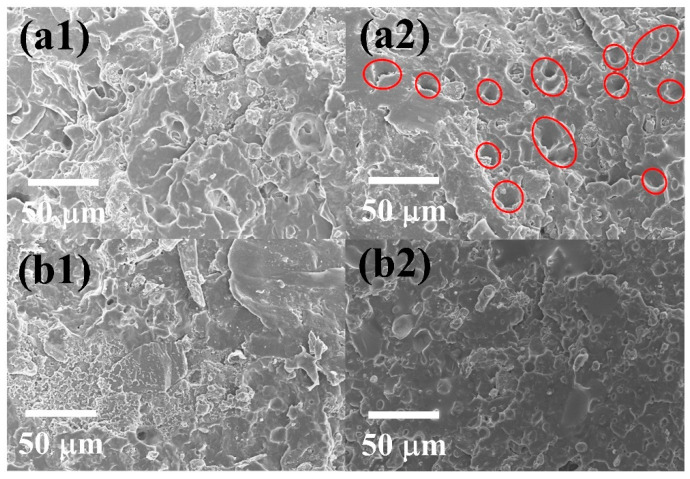
SEM images of the cryo-fractured surfaces of the (**a**) PP/APP/DPER and (**b**) PP/APP@PD/DPER composites before and after the water immersion. (“**1**”: before, “**2**”: after).

The cryo-fractured surface of PP composites was investigated by SEM to reveal the immigration of additives before and after water immersion, as shown in Figure 8. The PP/APP/DPER composite had numerous holes after water immersion, which proved that some APP had immigrated out of the composite. In contrast, the surface of the PP/APP@PD/DPER composite manifested much fewer holes; thus, APP@PD immigration was apparently limited.

**Figure 9:**
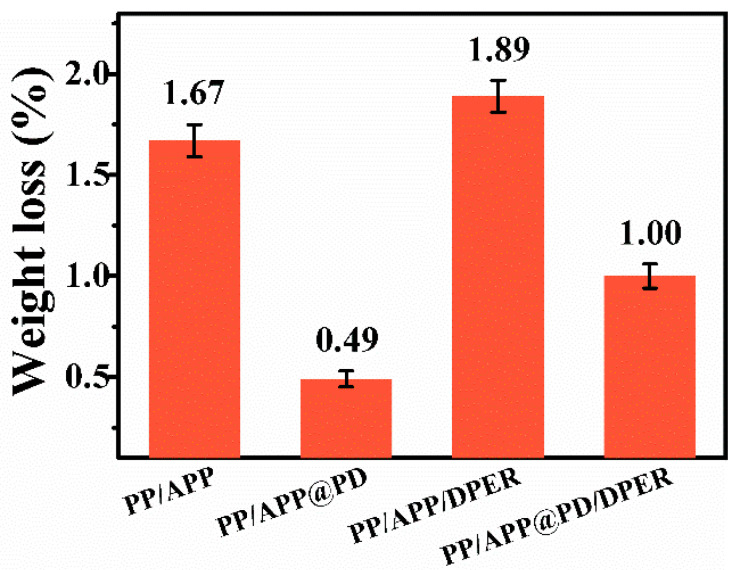
Weight loss of the PP/APP, PP/APP@PD, PP/APP/DPER, and PP/APP@PD/DPER composites with 25 wt.% additive loading after the water immersion at 70 °C for 168 h.

Furthermore, the variations in the weight loss of the PP composites before and after the water immersion test are listed in Figure 9. PP/APP/DPER and PP/APP displayed higher weight loss than that of PP/APP@PD/DPER and PP/APP@PD, representing better water resistance in APP@PD. In addition, it cannot be ignored that DPER-containing composites showed a greater weight loss, indicating the obvious migration of DPER during the water immersion process.

### 3.5. Mechanical Performance of the PP Composites

**Figure 10:**
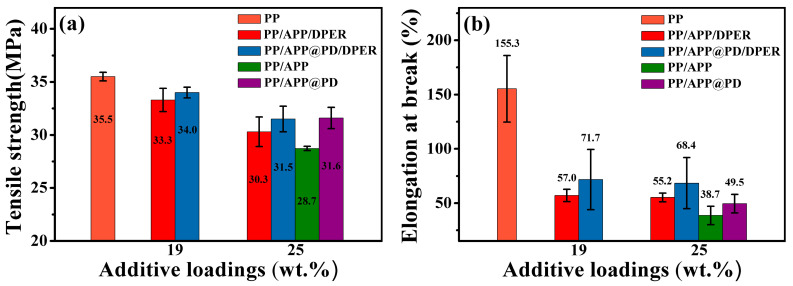
Tensile strength (**a**) and elongation at break (**b**) of the PP, PP/APP, PP/APP@PD, PP/APP/DPER, and PP/APP@PD/DPER composites.

The mechanical performance of PP/APP and PP/APP@PD composites was also tested for comparison to eliminate the influence of DPER. The results of tensile strength and elongation at break for PP and its composites are shown in Figure 10. The tensile strength of PP/APP@PD was 2.9 MPa higher than that of PP/APP, showing an obvious elevation, and the elongation at break results were better as well. The PP/APP@PD/DPER composites also reflected better tensile properties than the PP/APP/DPER composites. Hence, the incorporation of APP@PD apparently improved the tensile properties of PP flame retardant composites. The flexible chain of PD and good compatibility between APP@PD and PP are responsible for the increased tensile strength and elongation at break.

**Figure 11:**
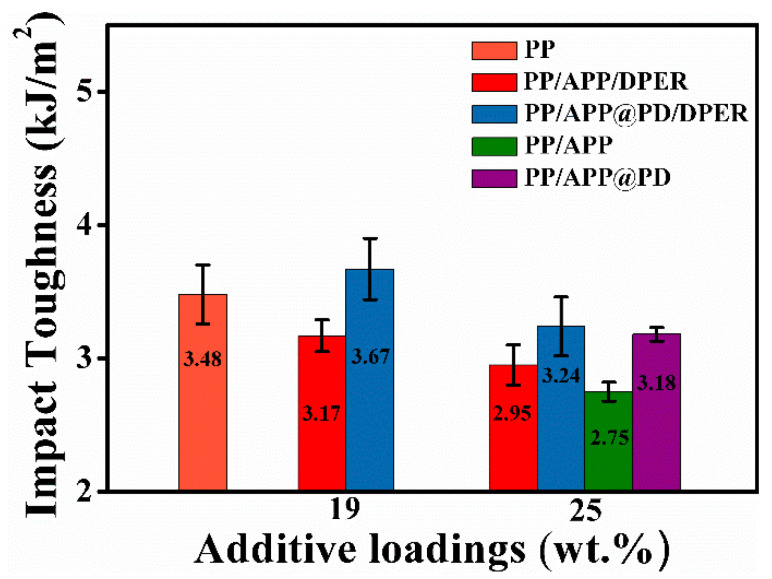
Impact toughness of the PP, PP/APP, PP/APP@PD, PP/APP/DPER, and PP/APP@PD/DPER composites.

To further study the influence of APP@PD on the toughness of PP composites, the notched Izod impact strengths of the composites were tested. As shown in Figure 11, the impact strength of pure PP was 3.48 kJ/m^2^, while that of PP/APP was 2.75 kJ/m^2^, reflecting a visible deterioration of the performance. However, the impact strength of PP/APP@PD was 3.18 kJ/m^2^, and this reinforcement was also found in the PP/APP@PD/DPER. More importantly, when the flame-retardant dosage was reduced to 19 wt.%, the impact strength of PP/APP@PD/DPER was 3.67 kJ/m^2^, which was higher than that of PP, explaining the improved toughness of the composite. Therefore, it is notable that APP@PD appeared to simultaneously improve the tensile, impact, and flame-retardant performances of the PP composites.

### 3.6. Interfacial Interaction between APP and PP before and after the Coating

It has been generally agreed in theory that secondary forces and hydrogen bonding are sufficient to produce adhesion between polymers to join themselves without the existence of chemical bonds [40]. Thus, the interfacial interaction between two materials influences the strength of their blends. To characterize the compatibility and interfacial interaction between APP@PD and PP matrix, which has been greatly improved, a method was adopted to calculate the interfacial free energy (IFE) between APP@PD and PP to ascertain their good compatibility quantitatively, which is called geometric-mean equations [41], as follows:

(1) $$\gamma_{a-b}=\gamma_{a}+\gamma_{b}-2\sqrt{\gamma_{a}^{d}\gamma_{b}^{d}}-2\sqrt{\gamma_{a}^{p}\gamma_{b}^{p}}$$

where $\gamma_{a-b}$ is the IFE between material a and b. $\gamma_{a}$ and $\gamma_{b}$ are the surface free energy (SFE) of material a and b, $\gamma_{a}^{d}$ and $\gamma_{b}^{d}$ are the dispersive components of SFE of material a and b, $\gamma_{a}^{p}$ and $\gamma_{b}^{p}$ are the polar components of SFE of material a and b. Generally, a low IFE between two materials implies good compatibility between them.

The surface free energy (SFE) of APP and PP were measured by the Owens–Wendt method [40], as follows:

(2) $$\gamma =\gamma^{d}+\gamma^{p}$$

(3) $$\gamma_{\mathrm{SL}}=\gamma_{S}+\gamma_{L}-2\sqrt{\gamma_{S}^{d}\gamma_{L}^{d}}-2\sqrt{\gamma_{S}^{p}\gamma_{L}^{p}}$$

where γ is the total SFE, $\gamma^{d}$ and $\gamma^{p}$ are the dispersive and polar parts of SFE, respectively. $\gamma_{S}$ and $\gamma_{L}$ are the SFE of solid and liquid materials, $\gamma_{S}^{d}$ and $\gamma_{L}^{d}$ are the dispersive components of SFE, $\gamma_{S}^{p}$ and $\gamma_{L}^{p}$ are the polar components of SFE, and $\gamma_{\mathrm{SL}}$ is the interfacial free energy between solid and liquid materials. According to the well-accepted Young’s equation:

(4) $$\gamma_{S}=\gamma_{L}\cos\theta \;+\gamma_{\mathrm{SL}}$$

where θ is the contact angle of a liquid material on a solid surface. Combining Equation (3) with (4), we get an equation:

(5) $$\frac{\left(1\;+\;\cos\theta \right)}{2}{\;\gamma }_{L}=\sqrt{\gamma_{S}^{d}{\;\gamma }_{L}^{d}}+\sqrt{\gamma_{S}^{p}\gamma_{L}^{p}}$$

Therefore, by using two liquids with known dispersive and polar parts, we can determine the SFE and its components in solid materials. Here, water and diiodomethane (DIM) were chosen as two test liquids. According to the literature [42], $\gamma_{L}^{d}$, $\gamma_{L}^{p}$, $\gamma_{L}$ are 21.8, 51.0, 72.8 mJ·m^−2^ of water and 50.8, 0, 50.8 mJ·m^−2^ of DIM, respectively. Herein, when these data are introduced into Equation (4), it can be simplified as:

(6) $$\gamma_{S}^{d}=12.7\;\times {\left(1\;+\cos\theta_{\mathrm{DIM}}\right)}^{2}$$

Therefore, the $\gamma_{S}^{d}$ can be obtained once the DIM contact angle is tested. Then, the Equation (5) follows that:

(7) $$\gamma_{S}^{p}={\left(5.097\;-\;0.654\sqrt{\gamma_{S}^{d}}+\;5.097\cos\theta_{\mathrm{water}}\right)}^{2}$$

Thus, the $\gamma_{S}^{d}$ is acquired when the water contact angle is measured. Finally, the SFE of the testing materials is found by Equation (8):

(8) $$\gamma_{S}=\gamma_{S\;}^{d}+\gamma_{S}^{p}$$

**Figure 12:**
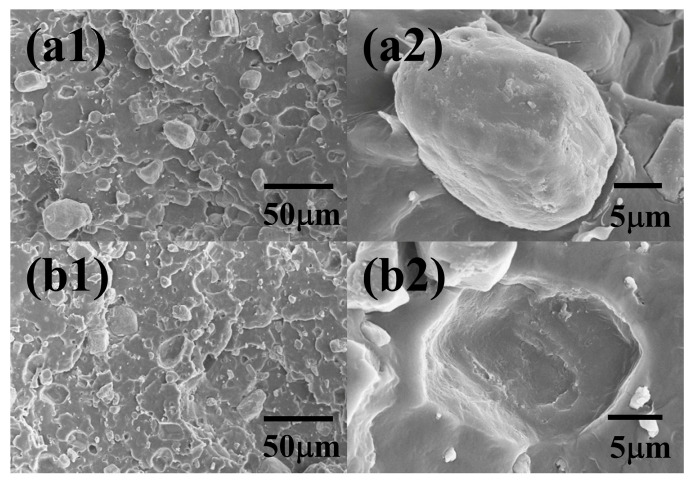
Surface morphologies of the PP/APP (**a**) and PP/APP@PD (**b**) composites after notched Izod impact test.

The water and DIM contact angles of PP, APP, and APP@PD are displayed in Figure S5, and the IFE between the PP matrix and APP or APP@PD is shown in Table 3. $\gamma_{\mathrm{PP}-\mathrm{APP}}$ was 15.66 mJ·m^−2^, while $\gamma_{\mathrm{PP}-\mathrm{APP}@\mathrm{PD}}$ was only 2.48 mJ·m^−2^, elucidating an 84.2% decrease and reinforcing interfacial interaction. Furthermore, the surfaces after impact testing of the composites confirmed this result (Figure 12). Some interfacial defects between the APP and the matrix were clearly observed, indicating poor compatibility with the matrix (Figure 12(a2)). However, the interface between APP@PD and PP became smoother (Figure 12(b2)), indicating improved interfacial interaction. In addition, as shown by the EDS mapping data in Figure S6, the particle dispersion of PP/APP@PD was more uniform than PP/APP, which benefited the flame retardancy and mechanical performance of the composites.

**Scheme 4:**
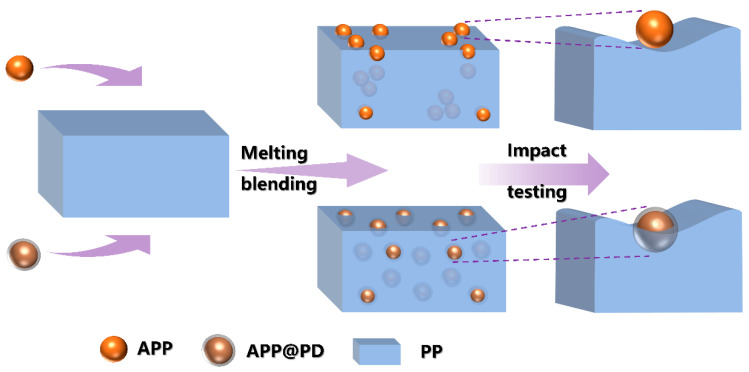
Schematic illustration of the interfacial interaction of APP and APP@PD in PP matrix.

On the basis of the above results, we propose the possible toughness mechanism of APP@PD for PP. As reflected in Scheme 4, during the melting and blending of the composites, the intrinsic high polarity of APP definitely inclined to an aggregation in the low-polarity PP matrix. After the impact test, APP preferred to be exposed on the substrate surface rather than embedded in it, representing its lower toughness. In contrast, the low polarity of the PD polymer segment in APP@PD contributed to its high dispersion and compatibility in the PP matrix, resulting in a high interfacial adhesion force between them, and ultimately dissipating more energy during the impact test.

## 4. Conclusions

APP was coated with a vinyl polysiloxane (PD) to improve its flame retardancy, wettability, and compatibility with PP. The good flame retardancy of PP/APP@PD/DPER ensured the V-0 rating at 19 wt.% IFR loading before water treatment and at 25 wt.% IFR loading after water treatment. Meanwhile, the decrease in PHRR, TSP, and delay of T_PSPR_ in the CCTs for PP/APP@PD/DPER proved the enhanced flame and smoke inhibition effect. The tensile strength, elongation at break, and impact strength were all improved compared to PP/APP/DPER. The interfacial free energy (IFE) between APP@PD and PP was calculated to evaluate the interfacial interaction between PP and APP@PD. The lower IFE between APP@PD and PP accounted for the improved compatibility and enhanced flame retardancy and mechanical properties of the composites. This study proposes a facile and unique coating technique for APP to enhance the flame retardancy, moisture sensitivity, and mechanical properties of halogen-free flame-retardant PP or other polymer materials. It is cost-effective and easy to industrialize; therefore, it may have potential applications in the automotive, electronic, and other fields. Nevertheless, it cannot be ignored that there was a visible decrease in the LOI of the composites after water immersion. Therefore, it is still urgent to further modify the surface of APP or explore new IFR systems to achieve high water resistance as well as flame retardancy in PP composites.

## Figures and Tables

**Table 1:** LOI and UL 94 results of the PP composites before and after the water immersion test at 70 °C for 168 h.

Sample	Untreated		After Water Treatment		
	LOI (%)	UL-94	LOI (%)	ΔLOI ^a^	UL-94
PP	18.7 ± 0.1	NR ^b^	18.7 ± 0.1	0	NR
PP/25APP	21.8 ± 0.3	NR	21.2 ± 0.2	2.8	NR
PP/25APP@PD	27.1 ± 0.2	NR	26.7 ± 0.2	1.5	NR
PP/25(APP/DPER)	29.6 ± 0.2	V-0	26.0 ± 0.2	12.2	NR
PP/25(APP@PD/DPER)	31.7 ± 0.3	V-0	28.3 ± 0.2	10.7	V-0
PP/19(APP/DPER)	26.3 ± 0.3	NR	24.0 ± 0.3	8.7	NR
PP/19(APP@PD/DPER)	28.3 ± 0.2	V-0	26.4 ± 0.2	6.7	NR

Note: ^a^ ΔLOI = (LOI_before_ − LOI_after_)/LOI_before_; ^b^ “NR” means no rating.

**Table 2:** TGA and DTG data of PP and its PP composites under nitrogen atmosphere.

Sample	T_d,5%_ (°C)	T_max_ (°C)	Residue (wt.%)		
			600 °C	700 °C	800 °C
PP	436	491	0.1	0.1	0.1
PP/APP/DPER	395	502	9.3	7.4	5.7
PP/APP@PD/DPER	397	499	9.6	9.0	8.4

**Table 3:** Surface free energy of APP and APP@PD and their interfacial free energy with PP.

Sample	$\gamma_{S}^{p}$ (mJ·m^−2^)	$\gamma_{S}^{d}$ (mJ·m^−2^)	γ (mJ·m^−2^)	$\gamma_{\mathrm{PP}-\mathrm{APP}}$ (mJ·m^−2^)
PP	2.95	26.28	29.23	
APP	29.89	40.84	70.73	15.66
APP@PD	0.02	26.50	26.52	2.48

## Data Availability

The data are available on request from the corresponding author.

## Supplementary Materials

The following supporting information can be downloaded at: https://www.mdpi.com/article/10.3390/polym15092074/s1. Table S1: Formulas of the PP composites; Table S2: Surface elemental content of APP@PD tested by XPS; Table S3: Cone calorimeter data of PP and its composites; Figure S1: (a) FTIR spectra of PD and D4-VI; (b) TGA and DTG curves of PD; Figure S2: Dispersion of APP and APP@PD in water after resting for 1 h; Figure S3: Digital photos of the residue of PP (a), PP/APP/DPER (b), and PP/APP@PD/DPER (c) after CCT (1: side view, 2: top view); Figure S4: FTIR spectra of char residues of PP/APP/DEPR, PP/APP/PD/DPER, and PP/APP@PD/DPER composites; Figure S5: Water and diiodomethane contact angle of PP, APP, and APP@PD; Figure S6: Images of SEM and EDS mapping of the PP/APP@PD (a) and PP/APP (b) composites after the brittle fracture treatment with liquid nitrogen.
