# Supplementary material for: Simultaneously Enhancing the Flame Retardancy, Water Resistance, and Mechanical Properties of Flame-Retardant Polypropylene via a Linear Vinyl Polysiloxane-Coated Ammonium Polyphosphate
Source: Polymers (Basel). 2023 Apr 27;15(9):2074. doi: 10.3390/polym15092074 (PMC10181116; doi:10.3390/polym15092074)
Supplement: Supplementary file 1 [file polymers-15-02074-s001.zip › polymers-2356503-supplementary.pdf]

Supplementary Materials for

Simultaneously enhancing the flame retardancy, water resistance, and mechanical properties of flame-retardant polypropylene via a linear vinyl polysiloxane coating ammonium polyphosphate

Qining Ke[1,2], Junchen Bai[1,2], Ge Zhang[1,2], Jiacheng Zhang[1,2], Mingshu Yang [1,2*]

[1] *Beijing National Laboratory for Molecular Sciences, Key Laboratory of Engineering Plastic, Institute of Chemistry, Chinese Academy of Sciences, Beijing, China.*

[2] *University of Chinese Academy of Sciences, Beijing, China.*

*Corresponding author: Mingshu Yang, E-mail: yms@iccas.ac.cn.

Table S1 Formulas of the PP composites.

Sample	PP (wt.%)	APP(@PD) [a] (wt.%)	DPER (wt.%)	PD (wt.%)	B215[b] (wt.%)
PP	99.7	0	0	0	0.3
PP/25APP	74.7	25	0	0	0.3
PP/25APP@PD	74.7	25	0	0	0.3
PP/25(APP/DPER)	74.7	20.0	5.0	0	0.3
PP/25(APP@PD/DPER)	74.7	20.0	5.0	0	0.3
PP/19(APP/DPER)	80.7	15.2	3.8	0	0.3
PP/19(APP@PD/DPER)	80.7	15.2	3.8	0	0.3

Note: [a] APP or APP@PD, [b] B215 was consisted of 1010 and 168 with a ratio of 1:2.

Table S2 Surface elemental content of APP@PD tested by XPS.

Sample	C(atom%)	P (atom%)	O(atom%)	N(atom%)	Si(atom%)
APP	29.5	12.6	39.4	18.5	-
APP@PD	59.2	-	19.4	-	21.4

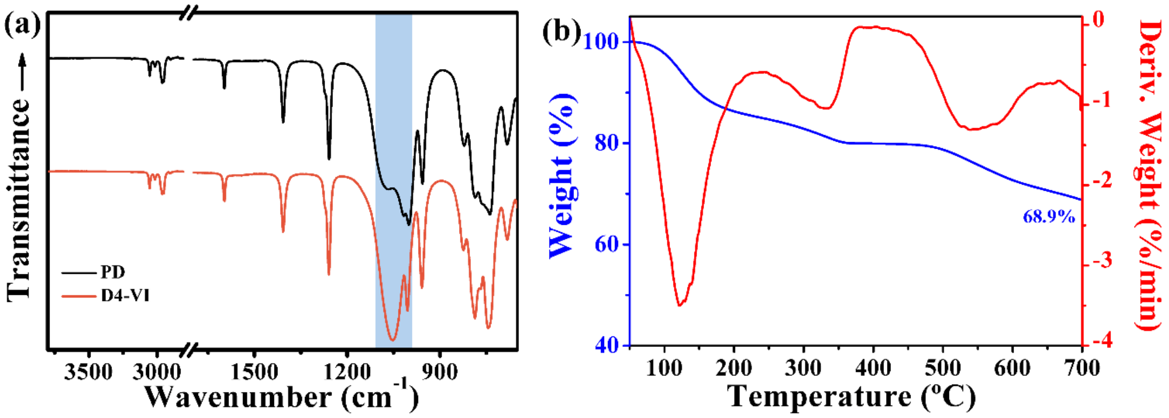

Figure S1. (a) FTIR spectra of PD and D4-VI; (b) TGA and DTG curves of PD.

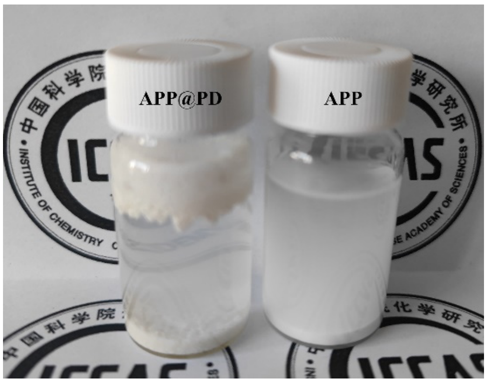

Figure S2. Dispersion of APP and APP@PD in water after resting for 1h.

Table S3 Cone calorimeter data of PP and its composites.

Sample	PP	PP/APP/DPER	PP/APP@PD/ DPER
TTI (s)	21	18	16
PHRR (kW·m^{-2})	783	412	304
THR (MJ·m^{-2})	92.9	84.5	84.2
av-EHC (MJ·kg^{-1})	42.5	35.4	37.3
TSP (m^{2})	11.5	18.2	15.2
PSPR (m^{2}·s^{-1})	0.093	0.088	0.073
T_{PSPR} (s)	120	205	390
Residue (wt.%)	0	9.4	12.3

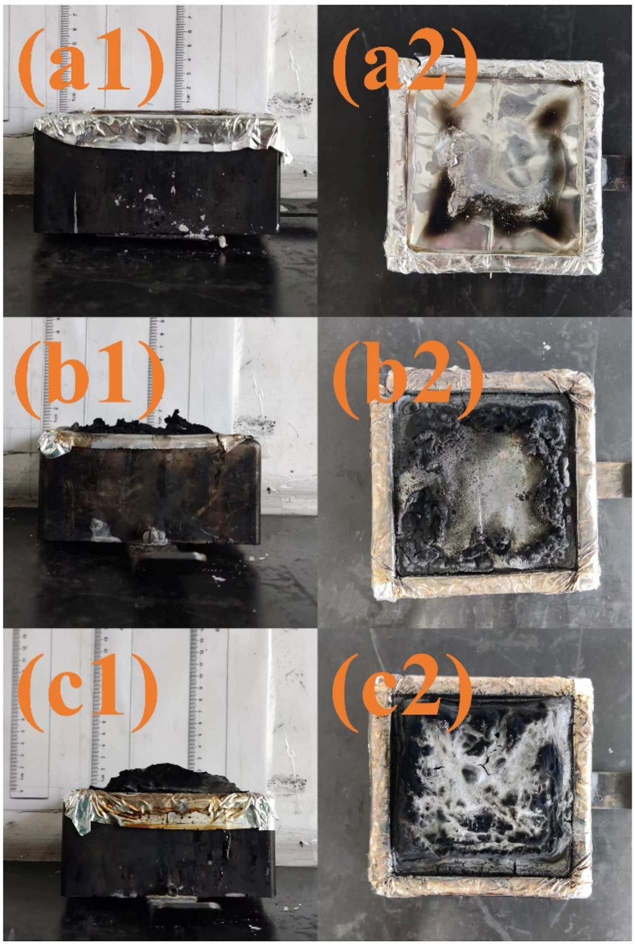

Figure S3. Digital photos of the residue of PP (a), PP/APP/DPER (b), and PP/APP@PD/DPER (c) after CCT. (1: side view, 2: top view)

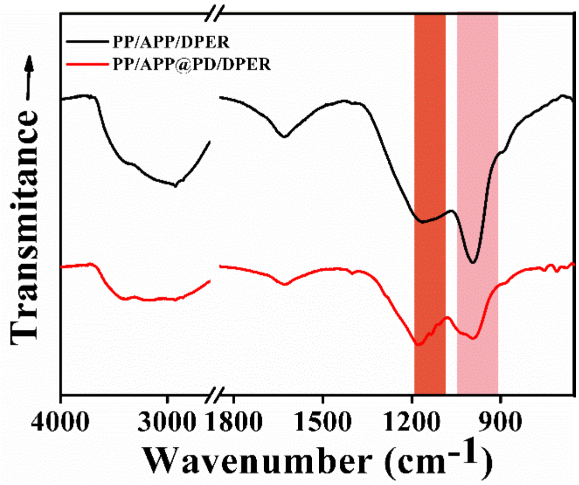

Figure S4. FTIR spectra of char residues of PP/APP/DEPR, PP/APP/PD/DPER, and PP/APP@PD/DPER composites.

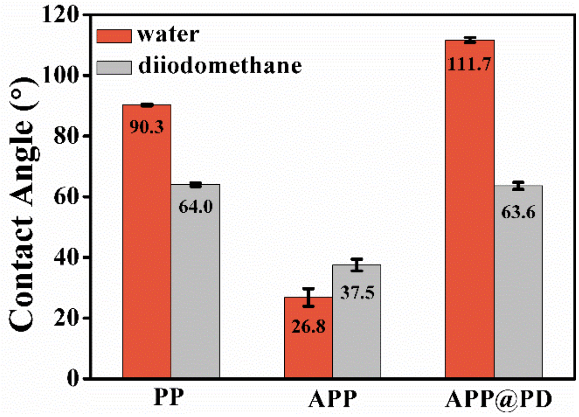

Figure S5. Water and diiodomethane contact angle of PP, APP, and APP@PD.

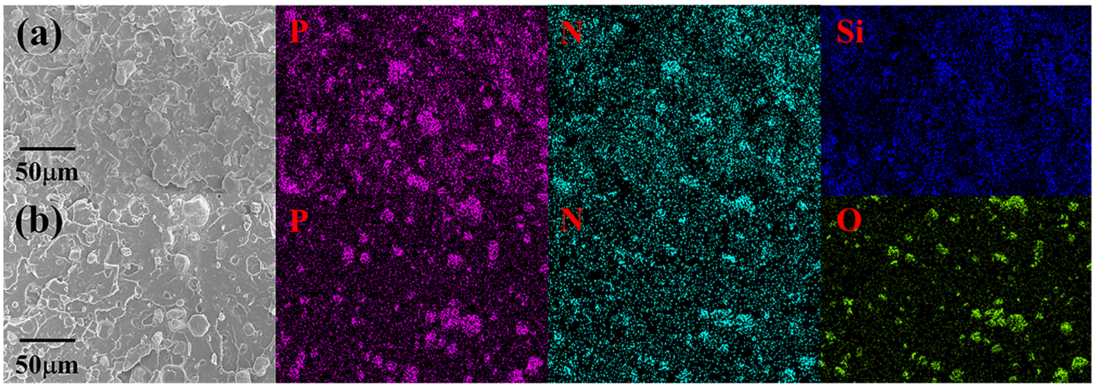

Figure S6. Images of SEM and EDS mapping of the PP/APP@PD (a) and PP/APP (b) composites after the brittle fracture treatment with liquid nitrogen.
